# Post-treatment status and unmet treatment needs after congenital heart defect screening policy in school-aged children: a multi-ethnic screening of 1.02 million in China

**DOI:** 10.3389/fpubh.2026.1778716

**Published:** 2026-04-29

**Authors:** Kai Bai, Yu Xia, Teng Wang, Shuyue Zhang, Lin Duo, Krishna Garu, Cai Kong, Lulu Zhang, Yuling Lu, Liping He, Qiuzhe Guo, Jiang Lu

**Affiliations:** 1 Fuwai Yunnan Hospital, Chinese Academy of Medical Science, Affiliated Cardiovascular Hospital of Kunming Medical University, Kunming, China; 2 School of Public Health, Kunming Medical University, Kunming, China; 3 Yunnan Population and Family Planning Research Institute, Wuhua District, Kunming, China

**Keywords:** CHD, community screening, multi-ethnic diversity, policy evaluation, prevalence, school-age children

## Abstract

**Objective:**

This large-scale, multi-ethnic screening of over 1.02 million school-aged children in China aimed to evaluate the real-world impact of recent congenital heart disease (CHD) screening policies by determining the community-based prevalence and clinical management status. Using a novel four-category framework (needing treatment, not needing treatment, postoperative, requiring follow-up), the study sought to identify persistent unmet needs and ethnic disparities in care access following policy implementation.

**Methods:**

Between 2024–2025, a cross-sectional screening of 1,024,531 school-aged children (3–18 years) was conducted across 12 ethnically diverse counties in Yunnan, Xinjiang and Chongqing, China. All participants underwent cardiac auscultation and confirmatory echocardiography. CHD cases were classified into one of four categories: Needing treatment, not needing treatment, postoperative, and follow-up. Multi-ethnic disparities in prevalence and defect types were analyzed.

**Results:**

A total of 3,376 children were diagnosed with CHD, yielding an overall prevalence of 3.30 per 1,000. Critically, the four-category stratification revealed: 48.7% were postoperative cases, 35.5% required no intervention, 14.3% needed treatment unmet, and 1.5% required further follow-up. Notably, substantial ethnic disparities were observed: Uyghurs had the highest postoperative rate (55%), whereas the Tujia minority group had the highest proportion of untreated but treatable cases.

**Conclusion:**

The findings revealed the achievement and underscored gaps in early diagnosis and equitable access to CHD care, particularly among ethnic minorities after CHD screening and care policy implementation, advocating for improved sustainability health policies to reduce unmet CHD needs in multi-ethnic populations.

## Background

Congenital disorders can be defined as structural or functional anomalies that occur during intrauterine life and claim an estimated 410,000 lives every year (1). Congenital heart disease (CHD) was one of the most common birth defects, affecting approximately 0.8% of live births worldwide (2) and CHD remained the leading cause of mortality among infants and young children (3). Actually, the global epidemiological data showed a prevalence of 3.809 per 1,000 among school-aged children (4) and it was estimated that a number of 11,998,283 people still lived with CHD, resulting in impaired quality of life (5).

In many developing countries, limitations in antenatal ultrasound screening and the frequent inconclusiveness of physical examination findings mean that many neonates with critical CHD are discharged without a timely and accurate diagnosis (6). Sudan and Iran CHD children (including school-age) prevalence was discovered to be 14.3 per 1,000 and 9.75% respectively, from hospital patients (7, 8). A systematic review further highlights this disparity in Africa, showing that the hospital-based prevalence of CHD in children and adolescents (12.63 per 1,000) was more than double the population-based estimate (5.12 per 1,000) (9).

In addition, Ethnic disparities in CHD are well-documented. Research from England and Wales (10) and the United States (11) indicates a disproportionately higher burden among certain ethnic groups. In China, studies have shown notable differences in CHD prevalence between minority populations and the Han majority (12), as well as variations in specific defect types (13).

Actually, China remained one of the countries with a significant burden of CHD. Qinghai Province reported the overall prevalence was 6.73% among school children (14). A high prevalence of 13.0 per 1,000 unrecognized CHD in school-age children was also identified in Yunnan, China (15). In response, the National Health Commission of China launched a nationwide neonatal CHD screening program in 2018 (16). Subsequently, Yunnan Province implemented the “Yunnan Provincial Congenital Heart Disease Screening Plan” to enhance early detection and intervention.

However, most existing data relied on hospital registries, which obscured the community prevalence and composition of CHD cases by clinical management status. A community-based prevalence encompassing the full spectrum of CHD—including cases requiring treatment, not requiring treatment, postoperative cases, and those needing regular follow-up—remains inadequately explored, especially among school-aged children in multi-ethnic regions after the policy was implemented. The study sought to evaluate the outcomes of the aforementioned health policies by revealing the current landscape of CHD, including treated cases and unresolved needs, thereby identifying the progress and the gaps in the healthcare system for school-aged children across diverse ethnic populations in China.

## Methods

### Study design and participants

Fuwai Yunnan Hospital conducted CHD screening for school-age children after the implementation of the nationwide neonatal CHD screening program and the “Yunnan Provincial Congenital Heart Disease Screening Plan” in 2018 and subsequently extended it to neighboring provinces. This cross-sectional study, conducted between June 2024 and June 2025, covered 12 counties/districts from 3 regions in China, including 6 counties (Jiangcheng, Jinggu, Zhengyuan, Jingdong, Mojiang, Qilin) in Yunnan Province; 3 counties (Akesu, Wensu, Awati) in Xinjiang Uyghur Autonomous Region; and 3 counties (Kaizhou, Yunyang, Xiushan) in Chongqing city.

The research focused on three regions characterized by high concentrations of ethnic minorities. The main ethnic minority in the Xinjiang Uyghur Autonomous Region was the Uyghur. In Chongqing Municipality, the Tujia ethnic group constitutes the primary minority population, whereas Yunnan Province was home to a diverse range of ethnic minorities, including the Hani, Yi, and Dai. These groups represent long-established, indigenous populations in their respective regions. Each of these groups possesses distinct health-related beliefs, practices, and socioeconomic contexts, which may significantly influence their health outcomes (17, 18). A total of 1,024,531 children and adolescents aged 3–18 years were included in the study.

The study population comprised all school-age children (3–18 years) enrolled in educational institutions within the selected screening regions, designed to be inclusive. All eligible children present during screening visits were enrolled, with rare exceptions (e.g., temporary absence). Exclusion criteria: Individuals were excluded from the study if they were diagnosed with, or found to have, acquired heart diseases (e.g., rheumatic heart disease, cardiomyopathy, infective endocarditis) during the screening echocardiographic examination.

### Data collection

The same screening team used the same type of echo with the same protocol. The screening team was divided into two groups, each staffed with at least one team leader, one cardiologist, one cardiac ultrasound physician, eight to ten cardiology nurses and a volunteer. Both groups operated concurrently to conduct the screenings. Basic information collection: Information on children by sex and age was collected by school teachers at the screening sites with the provincial document officially consent notice in advance.

First, each subject was physically examined by an experienced cardiologist, with special attention to growth and cyanosis, followed by cardiac auscultation, and for subjects with significant growth retardation, cyanosis, or pathologic murmurs, a cardiac sonographer used a portable ultrasound machine (Philips X50^®^) for examination (19). Volunteers recorded diagnoses according to ICD-10-CM (International Classification of Diseases, 10th Revision) (20); specific ICD-10-CM information is provided in the Supplementary material. Complex CHD included the following categories: severe pulmonary stenosis with significant sub-valvular obstruction and right-to-left interatrial shunting, tetralogy of Fallot, transposition of the great arteries with atrioventricular disharmony and ventriculo-arterial disharmony, and hypoplastic left heart syndrome. Cardiac ultrasonographers and cardiologists categorized children diagnosed with CHD based on the results of the examination. The categories were listed as follows: Not needing treatment: specific anatomical characteristics, including a Patent Foramen Ovale (PFO) with a resting diameter < 2 mm or an Atrial Septal Defect (ASD) with a diameter < 5 mm and an insufficient rim; absence of related symptoms (e.g., migraine, platypnea-orthodeoxia) or a history of paradoxical embolism; normal cardiopulmonary function without evidence of right ventricular volume overload; needing treatment: related symptoms (e.g., cryptogenic stroke, migraine, or dyspnea), objective evidence of right ventricular volume overload, or a clinically significant defect size (e.g., ASD with diameter≧5 mm); Postoperative: Patients who have received treatment (either thoracotomy or interventional procedures) and require subsequent follow-up are uniformly classified as postoperative cases for the purpose of this study; Regular follow-up was needed: It included patients who had a clear indication for surgical or interventional treatment, but in whom immediate intervention was contraindicated due to factors such as severe pulmonary hypertension. These patients required optimized medical therapy and intensive surveillance until their clinical condition became suitable for the procedure.

This study was approved by the Ethical Review Committee of Fuwai Yunnan Hospital (Approval No.2025–068-02). Informed consent was obtained from the legal guardians or parents of each enrolled child.

### Statistical analysis

Categorical variables were described by frequency and percentage (%). The prevalence of CHD and subtypes was expressed per 1,000 people. Differences in the prevalence of CHD in different regions were analyzed using the chi-square test or Fisher’s exact test. The characteristics of CHD patients were described according to diverse ethnic groups. Between-group comparisons used chi-square or Fisher’s exact tests for categorical variables, and Analysis of Variance (ANOVA) for continuous variables. We reported the prevalence of congenital heart disease (CHD), stratified by gender, age group, and geographic region. Furthermore, it delineates the patterns of ultrasound diagnosis and the distribution of specific CHD types across these diverse geographic and ethnic populations. In addition, a multinomial logistic regression model was employed to examine the associations of age, sex, region, and ethnicity with Treatment Demand outcomes in children with CHD, using the postoperative status as the reference category. Analyses were conducted using R software^®^ (version 4.4.3). A two-sided *p*-value of less than 0.05 was considered statistically significant.

## Results

### CHD screening and prevalence in 3 different regions

A total of 1,024,531 children aged 3–18 years were screened from 642 schools in Yunnan, 865 from Chongqing, and 380 from Xinjiang. A total of 3,376 children with CHD were identified, while 1,142 CHD out of 318,393 children (3.59 per 1,000). in Yunnan, 1,072 CHD out of 448,456 children (2.93 per 1,000), in Chongqing, 1,162 CHD out of 257,682 children (4.51 per 1,000), in Xinjiang (Supplementary Table S1; Supplementary Figure S1). A statistically significant variation in the prevalence of CHD was observed in the three regions, with the highest prevalence recorded in Xinjiang (4.51‰) and the lowest in Chongqing (2.93‰). In the distribution of CHD prevalence by sex and age groups in the three regions, Girls showed higher CHD prevalence than boys; children >12 years of age had the lowest prevalence compared to other age groups (Table 1).

**Table 1:** Prevalence and gender and age distribution of congenital heart disease (CHD) in 3 different regions.

	Yunnan (*n* = 318,393)			Chongqing (*n* = 448,456)			Xinjiang (*n* = 257,682)			*p* value
Age, year	*n*	CHD	Per 1,000 (95‰ CI)	*n*	CHD	Per 1,000 (95‰CI)	*n*	CHD	Per 1,000 (95‰ CI)	
Boys										
<6	21,941	78	3.55 (2.81, 4.43)	28,025	98	3.50 (2.84, 4.26)	10,160	42	4.13 (2.98, 5.58)	0.64
6–12	95,147	319	3.35 (3.00, 3.74)	111,678	252	2.26 (1.99, 2.55)	74,700	360	4.82^#^ (4.34, 5.34)	<0.01
>12	45,851	125	2.73 (2.27, 3.25)	95,189	157	1.65* (1.40, 1.93)	45,152	155	3.43 (2.91, 4.02)	<0.01
*P* value			0.09			<0.01			<0.01	
Girls										
<6	20,313	84	4.14 (3.30, 5.12)	25,422	87	3.42 (2.74, 4.22)	9,161	48	5.24 (3.87, 6.94)	0.06
6–12	89,159	384	4.31^#^ (3.89, 4.76)	101,735	299	2.94 (2.62,3.29)	73,872	392	5.31 (4.80, 5.86)	<0.01
>12	45,982	152	3.31 (2.80, 3.87)	86,407	179	2.07* (1.78, 2.40)	44,637	165	3.70* (3.15, 4.30)	<0.01
*P* value			<0.05			<0.01			<0.01	
Both										
<6	42,254	162	3.83 (3.27, 4.47)	53,447	185	3.46 (2.98, 4.00)	19,321	90	4.66 (3.75, 5.72)	0.07
6–12	184,306	703	3.81 (3.54, 4.11)	213,413	551	2.58 (2.37, 2.81)	148,572	752	5.06 (4.71, 5.44)	<0.01
>12	91,833	277	3.02* (2.67, 3.39)	181,596	336	1.85* (1.66, 2.06)	89,789	320	3.56* (3.18, 3.98)	<0.01
*P* value			<0.01			<0.01			<0.01	
Total	318,393	1,142	3.59* (3.38, 3.80)	448,456	1,072	2.39* (2.25, 2.54)	257,682	1,162	4.51* (4.25, 4.78)	<0.01

Statistical analysis was performed using the Pearson Chi-square test; *Compared with other groups, *p* < 0.05; ^#^Compared with >12 groups, *p* < 0.05.

### Characteristics of CHD patients of different ethnic groups in three regions

A total of 18 kinds of ethnic groups were identified among children with CHD and the majority of participants were Han, followed by Uygur, Tujia, Hani and Yi. Among them, 13 kinds of ethnic groups CHD found in Yunnan with 9 kinds as Unique ethnic groups and the Han, Hani, and Yi as the top three. There were 5 kinds of ethnic groups among CHD children in Chongqing, and the main ethnic groups were Han and Tujia. In Xinjiang, 7 kinds of ethnic groups were among CHD children, and the main ethnic groups were Uyghur and Han (Supplementary Figure S2). In total, 3,376 CHD children (1,586 boys and 1,790 girls) with a mean age of 10.03 ± 3.85 yrs. were included in the study. A statistically significant difference in the mean age of CHD children was observed with the smallest mean age of CHD children in Tujia (9.31 ± 4.16) (Table 2).

**Table 2:** Demographic characteristics of children with CHD from different ethnic groups.

Factors	Total	Han	Uygur	Tujia	Hani	Yi	Dai	Lahu	Miao	Others	*p* value
Age Mean ±SD [95CI]	10.03 ± 3.85 [9.90, 10.16]	9.81 ± 4.10 [9.62, 9.99]	10.53 ± 3.25 [10.32, 10.74]	9.31 ± 4.16 [8.66, 9.97]	10.36 ± 3.66 [9.78, 10.95]	9.64 ± 3.80 [9.01, 10.27]	9.92 ± 3.56 [8.91, 10.93]	10.14 ± 4.28 [8.48, 11.80]	10.88 ± 3.91 [9.30, 12.46]	11.15 ± 4.00 [9.54, 12.77]	<0.01^a^
Gender *N* (%) [95CI]											0.29^b^
Boys	1, 586 (47.0) [45.3, 48.7]	848 (46.3) [44.0, 48.6]	471 (48.9) [45.7, 52.1]	83 (52.9) [45.0, 60.7]	67 (44.4) [36.4, 52.6]	68 (47.9) [39.6, 56.2]	20 (40.0) [26.4, 53.6]	9 (32.1) [15.9, 48.3]	9 (34.6) [16.3, 52.9]	11 (42.3) [23.4, 61.2]	
Girls	1790 (53.0) [51.3, 54.7]	984 (53.7) [51.4, 56.0]	493 (51.1) [47.9, 54.3]	74 (47.1) [39.3, 50.0]	84 (55.6) [47.4, 53.6]	74 (52.1) [43.8, 60.4]	30 (60.0) [46.4, 73.6]	19 (67.9) [57.1, 84.1]	17 (65.4) [47.1, 83.7]	15 (57.7) [38.8, 76.6]	
Region *N* (%) [95CI]											<0.01^c^
Yunnan	1, 142 (33.8) [32.2, 35.6]	751 (41.0) [38.7, 43.3]	0 (0.0) [0.0, 0.0]	0 (0.0) [0.0, 0.0]	151 (100.0) [100.0, 100.0]	141 (99.3) [98.0, 99.6]	50 (100.0) [100.0, 100.0]	28 (100.0) [100.0, 100.0]	3 (11.5) [2.4, 30.2]	18 (69.2) [48.1, 85.0]	
Chongqing	1, 072 (31.8) [30.2, 33.4]	895 (48.9) [46.6, 51.2]	0 (0.0) [0.0, 0.0]	154 (98.1) [96.1, 100.0]	0 (0.0) [0.0, 0.0]	0 (0.0) [0.0, 0.0]	0 (0.0) [0.0, 0.0]	0 (0.0) [0.0, 0.0]	21 (80.8) [60.0, 92.7]	2 (7.7) [0.9, 25.1]	
Xinjiang	1, 162 (34.4) [32.8, 36.0]	186 (10.1) [8.8, 11.6]	964 (100.0) [100.0, 100.0]	3 (1.9) [0.0, 3.9]	0 (0.0) [0.0, 0.0]	1 (0.7) [0.0, 1.4]	0 (0.0) [0.0, 0.0]	0 (0.0) [0.0, 0.0]	2 (7.7) [0.9, 25.1]	6 (23.1) [9.0, 43.7]	

^a^One-way ANOVA; ^b^Pearson Chi-square test; ^c^Fisher’s exact test.

### Ultrasound diagnosis of CHD in children of different ethnic groups in three regions

**Figure 1:**
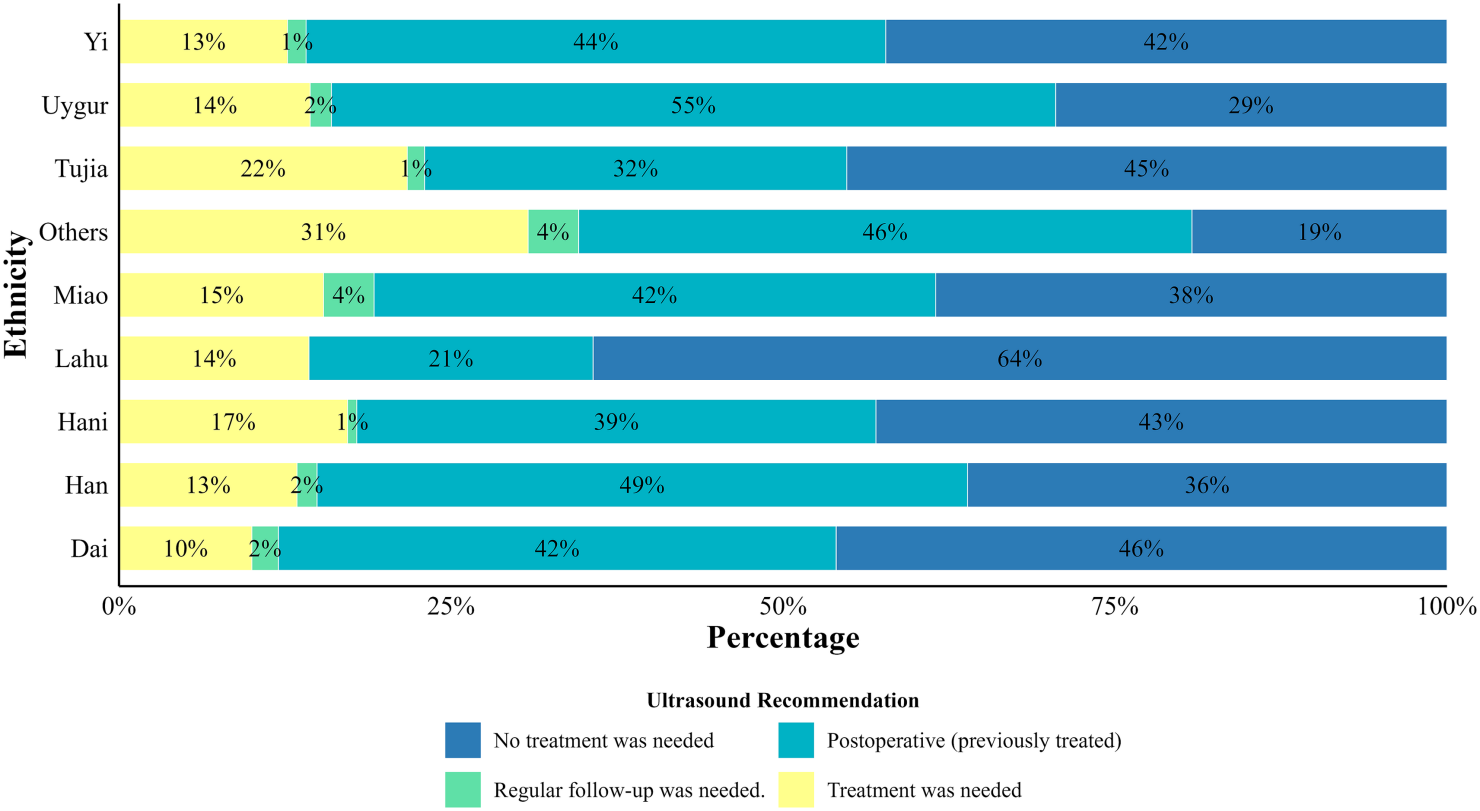
Ultrasound recommendations for children with CHD from different ethnic groups. Others: Ethnic groups with a population of fewer than 10 individuals include the Bai, Hui, Bulang, Jinuo, Zhuang, Gelao, Wa, Tibetan, Yao, and Lao Long.

**Figure 2:**
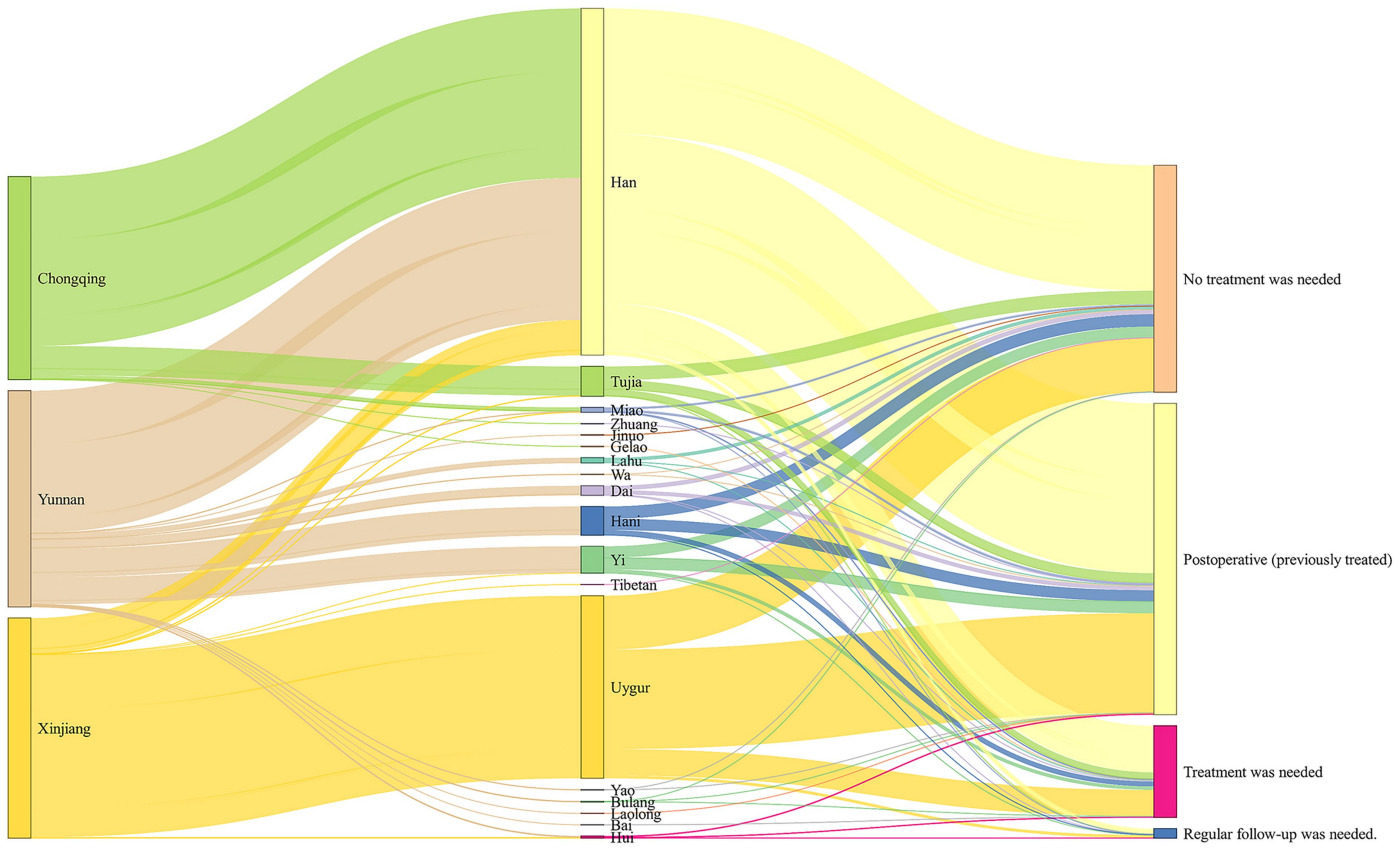
Ethnic groups and ultrasound examination recommendations flow at different regions. The Sankey diagram was applied to visualize the distribution of ethnic groups and the composition of ultrasound examination recommendations in different regions. The first column of bars indicates the region groups, the second column indicates the ethnic groups and the third column of bars indicates the ultrasound examination recommendation. This Sankey diagram represents the composition of the ethnic groups in different regions from left to right, and further flows into the ultrasound examination recommendations.

Figures 1, 2 showed ultrasound diagnosis of CHD prevalence in 4 kinds of status as needing treatment, not needing treatment, postoperative and need regular follow-up. Overall, the proportion of postoperative CHD was the highest (48.7%), and the proportion of patients requiring treatment was 14.3%. followed by CHD did not require treatment (35.5%) and needed regular follow-up (1.5%) (Supplementary Figures S3, S4). Figures 1, 2 also showed the distribution of CHD patients from different ethnic groups in 3 regions, the highest percentage of postoperative cases was among Uyghurs (55%), the highest percentage requiring treatment was among small number of other ethnic groups (31%), and the highest percentage not requiring treatment was among Lahu (64%) (Figure 1).

### The composition types of CHD by ethnicity in the 3 regions

**Figure 3:**
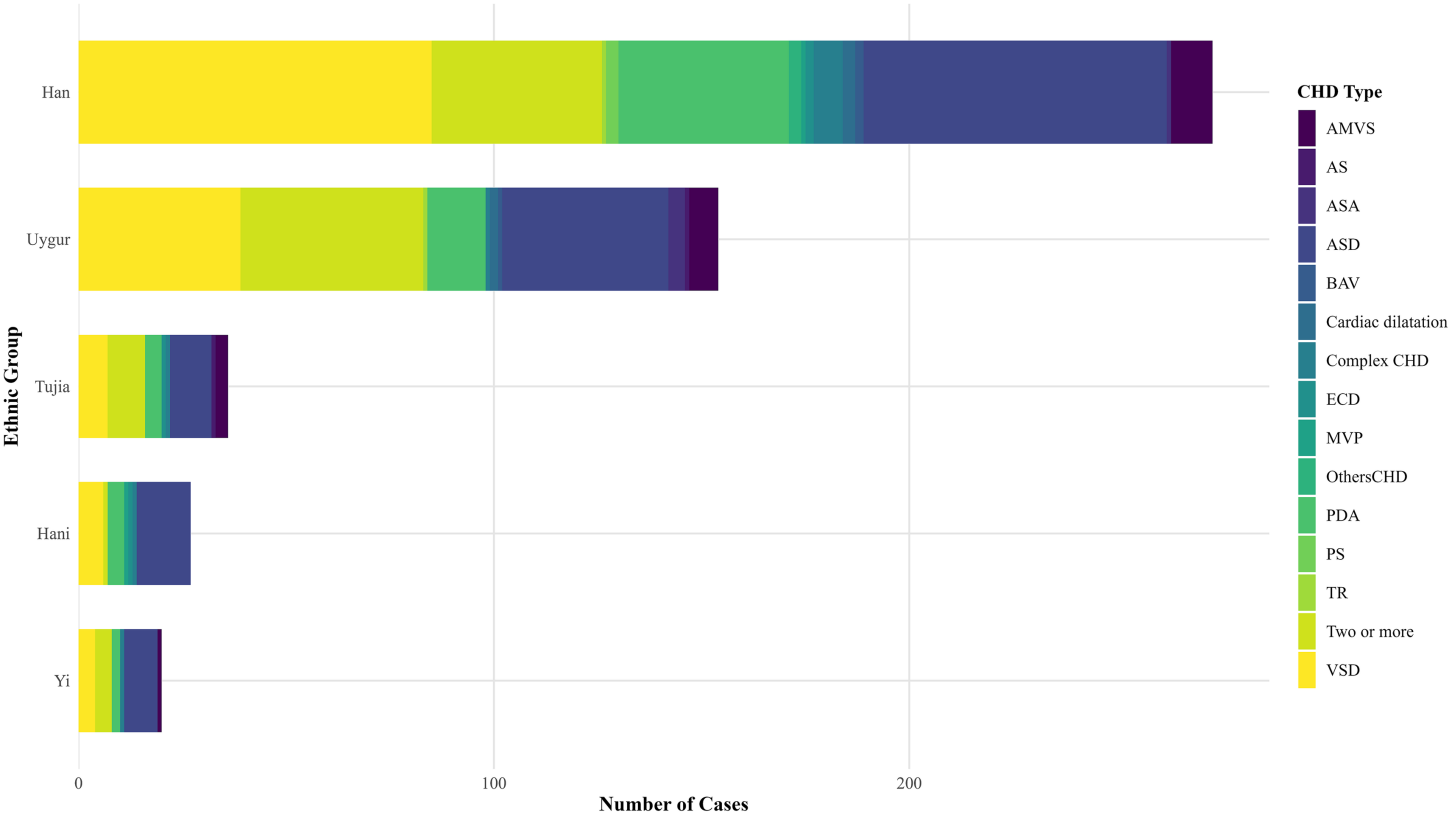
The distribution of CHD types among different ethnic groups among pediatric patients requiring treatment for CHD. The figure exclusively encompasses ethnic groups with a minimum of ten children diagnosed with CHD.

The distribution of types of needing treatment for CHD among different ethnic groups in school-age children showed that the four most prevalent CHD types were atrial septal defect (ASD), ventricular septal defect (VSD), patent ductus arteriosus (PDA), and two or more CHD types. The most prevalent CHD type varies among ethnic groups, as ASD was the most prevalent among the Hani, Tujia, and Yi ethnic groups, VSD among the Han ethnic group, and two or more CHD types among the Uyghur ethnic group (Figure 3; Supplementary Figure S5). The types of CHD (The exclusion of the absence of postoperative CHD diagnosis information) as VSD, PDA, and dextrocardia, were more prevalent in Chongqing than in Yunnan and Xinjiang. Concurrently, the coexistence of two or more different types of CHD coexisted at notably lower rates in Yunnan than in other regions (Table 3; Supplementary Figure S6).

**Table 3:** Distribution of CHD types requiring treatment among pediatric patients in the three regions.

CHD types *N* (%) [95CI]	Total	Yunnan	Chongqing	Xinjiang	*P* value
Patent foramen ovale (PFO)	508 (29.3) [27.2, 31.5]	182 (30.3) [26.8, 34.1]	151 (24.6) [21.3, 28.2]	175 (33.7) [29.8, 37.9]	<0.01
Atrial septal defect (ASD)	209 (12.1) [10.6, 13.7]	72 (12.0) [9.6, 14.9]	68 (11.1) [8.8, 13.8]	69 (13.3) [10.6, 16.5]	
Ventricular septal defect (VSD)	162 (9.3) [8.0, 10.8]	43 (7.2) [5.3, 9.5]	73 (11.9)* [9.5, 14.7]	46 (8.9) [6.7, 11.6]	
Bicuspid aortic valve (BAV)	113 (6.5) [5.4, 7.8]	44 (7.3) [5.4, 9.7]	40 (6.5) [4.8, 8.8]	29 (5.6) [3.9, 8.0]	
Patent ductus arteriosus (PDA)	80 (4.6) [3.7, 5.7]	28 (4.7)* [3.2, 6.7]	34 (5.5)* [3.9, 7.7]	18 (3.5)* [2.1, 5.5]	
Tricuspid Atresia (TA)	76 (4.4) [3.5, 5.5]	51 (8.5)* [6.5, 11.0]	13 (2.1) [1.2, 3.6]	12 (2.3) [1.2, 4.0]	
Pulmonary stenosis (PS)	72 (4.2) [3.3, 5.2]	29 (4.8) [3.3, 6.9]	30 (4.9) [3.4, 6.9]	13 (2.5)* [1.4, 4.3]	
Aortic stenosis (AS)	47 (2.7) [2.0, 3.6]	12 (2.0) [1.1, 3.5]	18 (2.9) [1.8, 4.6]	17 (3.3) [2.0, 5.2]	
Mitral valve prolapse (MVP)	40 (2.3) [1.7, 3.1]	20 (3.3) [2.1, 5.1]	9 (1.5) [0.7, 2.8]	11 (2.1) [1.1, 3.8]	
Atrial septal aneurysm (ASA)	39 (2.3) [1.6, 3.1]	22 (3.7)* [2.4, 5.5]	8 (1.3) [0.6, 2.6]	9 (1.7) [0.8, 3.3]	
Cardiac dilatation	36 (2.1) [1.5, 2.9]	13 (2.2) [1.2, 3.7]	11 (1.8) [0.9, 3.2]	12 (2.3) [1.2, 4.0]	
Aneurysm of the membranous ventricular septum (AMVS)	29 (1.7) [1.1, 2.4]	5 (0.8) [1.2, 3.7]	14 (2.3) [1.3, 3.8]	10 (1.9) [1.0, 3.5]	
Persistent left superior cava (PLSC)	12 (0.7) [0.4, 1.2]	4 (0.7)* [0.2, 1.7]	8 (1.3)* [0.6, 2.6]	0 (0.0)* [0.0, 0.7]	
Dextrocardia	11 (0.6) [0.3, 1.1]	1 (0.2) [0.0, 1.0]	9 (1.5)* [0.7, 2.8]	1 (0.2) [0.0, 1.1]	
Endocardial cushion defect (ECD)	5 (0.3) [0.1, 0.7]	3 (0.5) [0.1, 1.5]	2 (0.3) [0.0, 1.1]	0 (0.0) [0.0, 0.7]	
Others CHD	42 (2.4) [1.8, 3.3]	12 (2.0) [1.1, 3.5]	18 (2.9) [1.8, 4.6]	12 (2.3) [1.2, 4.0]	
Two or more types of CHD	239 (13.8) [12.2, 15.5]	53 (8.8)* [6.8, 11.3]	102 (16.6) [13.8, 19.8]	84 (16.2) [13.2, 19.6]	
Complex CHD	13 (0.8) [0.4, 1.3]	6 (1.0) [0.4, 2.1]	6 (1.0) [0.4, 2.1]	1 (0.2) [0.0, 1.1]	

Statistical analysis was performed using the Pearson Chi-square test; *Compared with other regions, *P* < 0.05. Others CHD: includes CHD types with fewer than 10 patients. Two or more types of CHD: includes two or more different types of CHD.

### Multinomial analysis of factors associated with treatment demand in Pediatric CHD

Table 4 presented the results of the multinomial logistic regression analysis. Compared to the postoperative reference group, older age was associated with lower odds of both requiring treatment (OR = 0.93, 95% CI: 0.91–0.96) and not needing treatment (OR = 0.87, 95% CI: 0.85–0.89). Boys had lower odds of requiring treatment than girls (OR = 0.68, 95% CI: 0.55–0.84). Compared to Yunnan, children from Chongqing had higher odds for both outcomes (Needing treatment: OR = 2.13, 95% CI: 1.56–2.91; Not needing treatment: OR = 1.26, 95% CI: 1.02–1.57). Among ethnic groups, Tujia, Hani, and Lahu children showed elevated odds ratios across both outcome categories compared to Han children.

**Table 4:** Factors associated with treatment demand among children with congenital heart disease: results from a multinomial logistic regression analysis.

Factors	Reference: Postoperative		
	Needing treatment	Not needing treatment	Regular follow-up was needed
	Exp β (95% C.I)	Exp β (95% C.I)	Exp β (95% C.I)
Age	0.93 (0.91, 0.96)***	0.87 (0.85, 0.89)***	1.06 (0.98, 1.15)
Gender (ref. Girls)			
Boys	0.68 (0.55, 0.84)***	1.06 (0.91, 1.24)	0.87 (0.50, 1.54)
Region (ref. Yunnan)			
Chongqing	2.13 (1.56, 2.91)***	1.26 (1.02, 1.57)*	1.04 (0.48, 2.27)
Xinjiang	1.27 (0.79, 2.06)	0.63 (0.43, 0.91)	0.24 (0.03, 1.86)
Ethnicity (ref. Han)			
Uygur	1.21 (0.76, 1.90)	1.36 (0.94, 1.98)	3.62 (0.47, 27.86)
Tujia	1.82 (1.13, 2.94)*	1.60 (1.07, 2.39)*	1.23 (0.27, 5.56)
Hani	2.54 (1.50, 4.32)**	1.77 (1.19, 2.63)**	0.51 (0.06, 3.96)
Yi	1.61 (0.90, 2.88)	1.40 (0.94, 2.08)	1.01 (0.22, 4.61)
Dai	1.30 (0.47, 3.57)	1.65 (0.88, 3.09)	1.45 (0.18, 11.67)
Lahu	3.76 (1.03, 13.74)*	5.02 (1.91, 13.17)**	**2.78 × 10** ^**–8#**^
Miao	1.11 (0.35, 3.57)	1.34 (0.55, 3.27)	2.70 (0.33, 22.03)
Others	3.50 (1.38, 8.86)**	0.77 (0.26, 2.24)	2.91 (0.35, 24.04)

*p < 0.05; **p < 0.01; ***p < 0.001; ^#^The extreme estimate for the Lahu group results from complete separation, rendering the OR and its CI incalculable. This estimate is presented for completeness but should not be interpreted substantively. Bold text indicates that the result is statistically significant.

## Discussion

This large-scale community-based screening program of CHD in school-aged children, involving 1.02 million children, provided a clinic status meaning prevalence stratified into four distinct clinical/management categories: those requiring treatment, not requiring treatment, postoperative cases, and those needing regular follow-up. Conducted in the context of significant national and provincial public health initiatives—including the National Health Commission’s nationwide neonatal CHD screening program launched in 2018 and subsequent “Yunnan Provincial Children’s Congenital Heart Disease Screening Plan”. It described the contemporary landscape of CHD in these communities, encompassing both cases that had received intervention and those with persistent unmet needs, thereby outlining the profile of care and gaps among school-aged children across diverse ethnic populations.

The manuscript revealed significant regional differences in CHD prevalence, with Xinjiang exhibiting the highest rate (4.51‰), followed by Yunnan (3.59‰), and Chongqing the lowest (2.39‰). These rates are notably lower than the global birth prevalence reported in a meta-analysis (9.1 per 1,000 live births) (21). However, the global CHD prevalence encompasses particularly severe and complex cases that are diagnosed within the first week or that result in early mortality. In contrast, our study represents a community-based cross-sectional screening. By design, this approach inherently missed those most severe cases that were absent from the school education or succumbed before the screening age. Consequently, our prevalence figure essentially reflects the prevalence of CHD surviving to the screening age within the community. This may result in inherently lower than the reported prevalence. Moreover, the discrepancy may reflect improvements in socioeconomic conditions, prenatal care, and reduced exposure to CHD risk factors in these regions over the past decade. The adoption of a healthy lifestyle in the survey areas and the popularization of knowledge of prenatal and postnatal care may have reduced the exposure of pregnant women to CHD risk factors during pregnancy (22–24), aligning with broader goals to reduce the CHD burden in low- and middle-income countries (25).

A central finding was the four-category stratification of CHD cases. The fact that 48.7% of identified children were postoperative cases suggested substantial success in policy implementation, which has achieved considerable coverage by the national and provincial screening policies, which improved early detection and care. This was in stark contrast to contexts with severe resource constraints, such as in sub-Saharan Africa, where insufficient availability of cardiac surgery services results in well under 3% of children in need actually receiving it (26). Moreover, the prevalence of treatable CHD in our study (0.5 treatable cases per 1,000 children) was substantially lower than the rate reported in a school-based screening in Cambodia and China (2017–2020), which identified 1.2 treatable cases per 1,000 children screened (27). However, the persistence of unrecognized, treatable CHD still highlighted a critical residual gap. Without timely postnatal diagnosis, these children transition into the community as “unmet need” school-age cases, constituting a continued and often overlooked disease burden.

Analysis across ethnic groups revealed distinct patterns in treatment access and outcomes, offering a view of policy effectiveness. The highest postoperative rate was observed among Uyghur children (55%), which may be attributed to targeted government-led initiatives, especially free screening and treatment programs in Xinjiang (28). This success demonstrated the potential of focused policies to achieve treatment rates commensurate with high-income countries (26). Conversely, persistent disparities were evident among other ethnic minorities. For instance, both the Tujia and Hani groups had significantly higher odds of needing treatment compared to the Han majority. Similar trends were observed among ethnic minorities with smaller sample sizes pooled into the “Others” category, such as Hui, Yao, and Bulang. While these disparities are described along ethnic lines, they likely reflect underlying structural and socioeconomic determinants rather than ethnicity per se. Potential explanatory factors may include greater average distance to tertiary cardiac centers, variations in local insurance coverage, differences in health literacy, or culturally influenced care-seeking behaviors. This pattern aligned with global trends where ethnic minorities often faced barriers to healthcare access (29). The children from ethnic minorities may encounter barriers to receiving a diagnosis and treatment due to their economic situation, accessibility of medical resources, and other factors (Supplementary Table S2) (30, 31). Therefore, it was imperative to direct particular attention to the issue of health resource distribution among ethnic minority children with CHD. This necessitates the development of policies aimed at reducing ethnic treatment disparities among CHD patients.

In additional, the CHD prevalence pattern exhibited that the most prevalent CHD types varied by ethnicity, as the Han Chinese constitute the predominant ethnic group in China, presented with higher proportion of ventricular septal defects (VSDs), while other ethnic minorities, including the Hani and Yi, as well as the Tujia, account for the largest proportion of atrial septal defects (ASD), Uyghur children had the highest proportion of complex CHD (two or more types), posing greater treatment challenges (5). These findings mirror ethnic-specific patterns observed in other studies (32–34), suggesting potential genetic, environmental, or cultural influences. Furthermore, a case–control study of Finnish origin revealed that maternal alcohol consumption during the first trimester of pregnancy appeared to double the risk of atrial septal defect (OR = 1.9, CI98% = 1.1–3.4) (35). The Hani, Yi, and Tujia ethnic groups were renowned for their drinking culture. To mitigate the burden of CHD among minority groups, it was imperative to disseminate information regarding the dangers of alcohol consumption and improve the health services for pregnant women.

In summary, this cross-sectional survey, conducted 6 years after the implementation of major CHD screening policies, provided a unique snapshot of their impact. It confirmed progress in increasing treatment rates (evident in the high postoperative proportion) but simultaneously uncovered a significant residual burden of undiagnosed, treatable disease and persistent ethnic disparities. The findings advocated not only for the continued support and expansion of school-based screening programs but also for more targeted, equity-focused strategies to reach underserved ethnic minority communities. Future policies must address the specific ethnic related barriers identified to transform screening success into universal treatment access and ultimately improve long-term outcomes for all children with CHD in China.

### Study limitations

First, despite the explanations provided in the discussion, the prevalence of CHD reported in this study should be considered a potential underestimate. Future studies should actively track children after delivery in the hospital and integrate community health records to better capture missed cases and refine prevalence estimates. Secondly, given the expansive nature of this study, which entailed a large-scale screening involving one million participants failed to collect personal information on all school-age children who participated in the survey. Consequently, the prevalence of CHD in various ethnic groups could not be ascertained. Furthermore, due to limitations in resources and study duration, this study was community-based rather than population-based; therefore, the findings from the districts involved in this study may not reflect the actual situation of the prevalence of CHD in Yunnan, Chongqing, and Xinjiang. Finally, the cross-sectional design provides only a snapshot and cannot distinguish screening-detected and self-referred postoperative cases, limiting causal attribution to screening policy. Actually, the achievements in detection and treatment to some extend resulted in high percentage of postoperative CHD reflected the influence of the policy. However, the persistent proportion of undiagnosed and needing treatment still revealed a clear unmet need and the gap of the policy remained in the community. Longitudinal population studies are needed to assess temporal trends, evaluate sustained policy impact, and identify specific gaps in the continuum from detection to treatment completion.

### Future directions

To address the identified gaps between screening and treatment, future initiatives should focus on integrating school-based screening with longitudinal clinical registries or referral tracking systems. This would allow real-time monitoring of children along the care continuum and improve treatment uptake. Additionally, implementation research is needed to identify and mitigate the structural, geographic, and cultural barriers that disproportionately affect underserved ethnic groups.

## Conclusion

This study documents both progress and gaps in congenital heart disease care within diverse communities. While a high proportion of postoperative cases reflects improved early detection, the persistence of undiagnosed, treatable disease reveals a clear unmet need and a discontinuity between screening and definitive treatment. Observed ethnic and regional disparities in prevalence and treatment access highlight unequal policy benefits, likely rooted in structural and socioeconomic factors rather than ethnicity itself. The proposed simplified CHD categorization offers a practical tool for community-level surveillance. Future success requires integrating sustained screening with longitudinal tracking and implementing equity-focused, culturally informed interventions for underserved populations.

## Data Availability

The raw data supporting the conclusions of this article will be made available by the authors, without undue reservation.
