# Supplementary material for: Post-treatment status and unmet treatment needs after congenital heart defect screening policy in school-aged children: a multi-ethnic screening of 1.02 million in China
Source: Front Public Health. 2026 Apr 29;14:1778716. doi: 10.3389/fpubh.2026.1778716 (PMC13168060; doi:10.3389/fpubh.2026.1778716)
Supplement: Supplementary file 1 [file Data_Sheet_1.docx]

Supplementary Material

**Supplementary Figures**


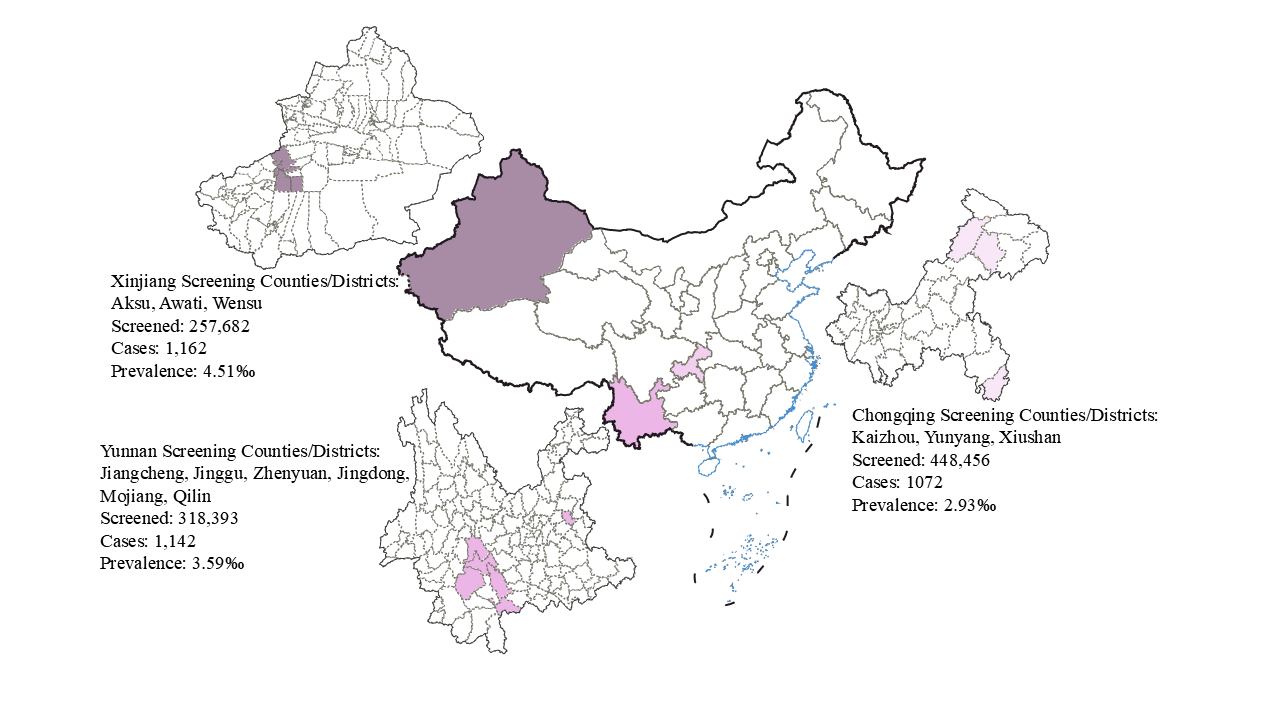


**Figure S1** Distribution Map of Congenital Heart Disease Screening Among School-Age Children in Three Regions of China.


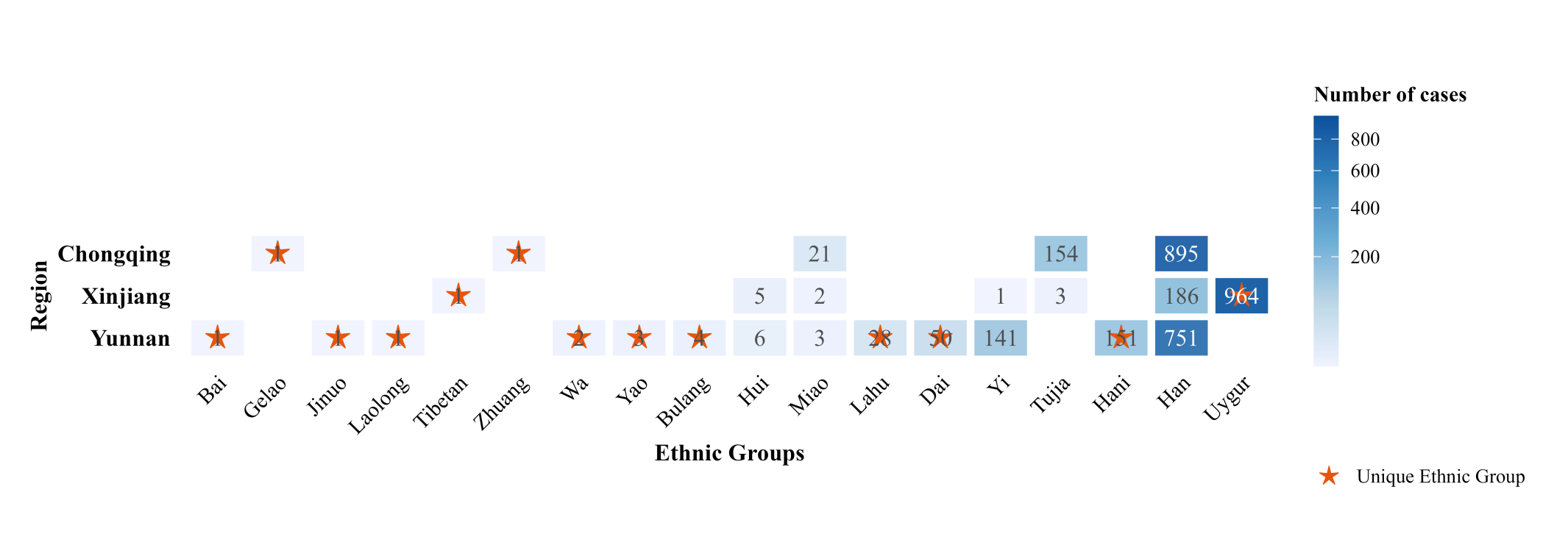


**Figure S2** Distribution of CHD patients by ethnic groups in three regions.


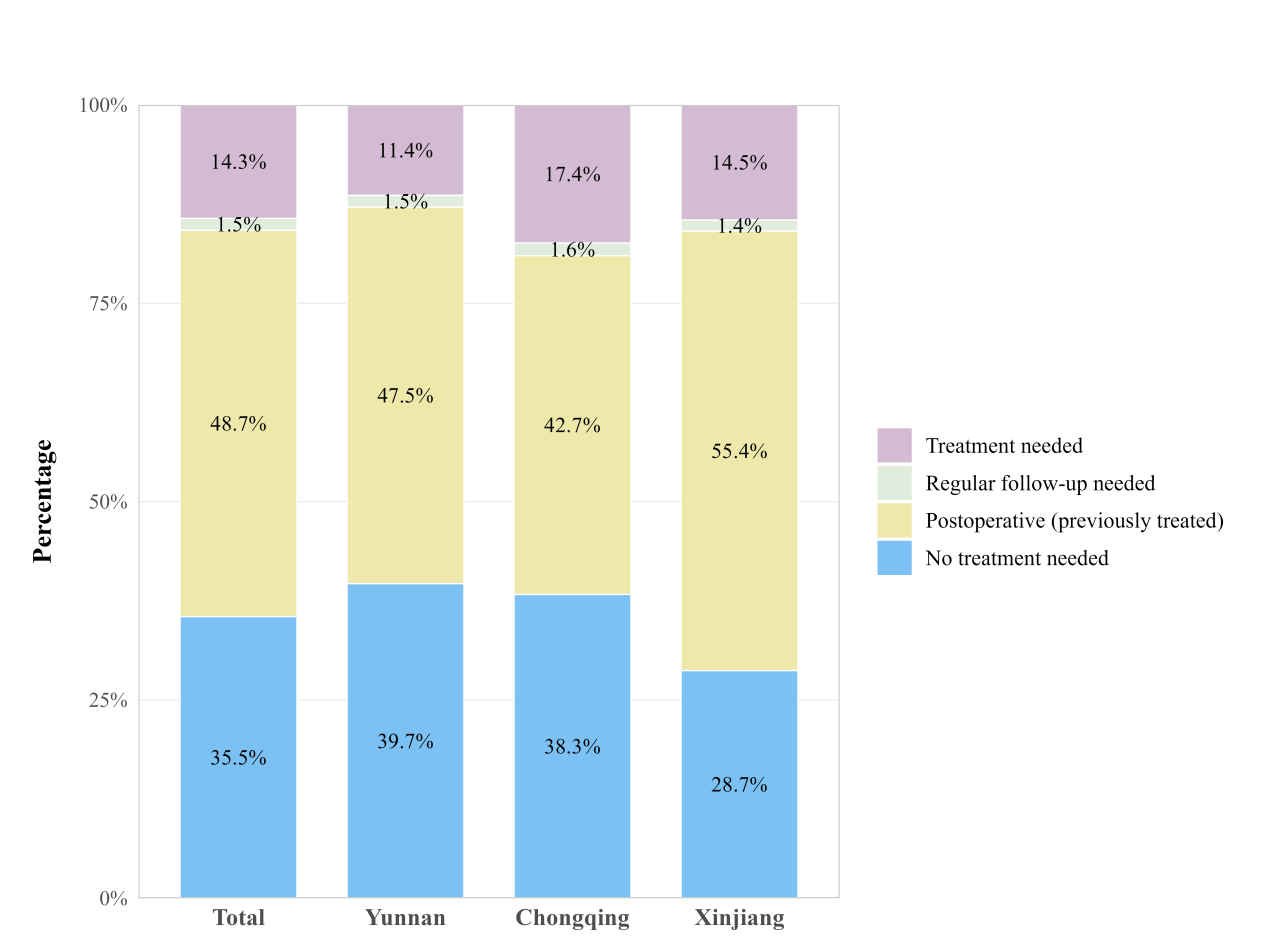


**Figure S3** Ultrasound examination recommendations for CHD children in three regions.


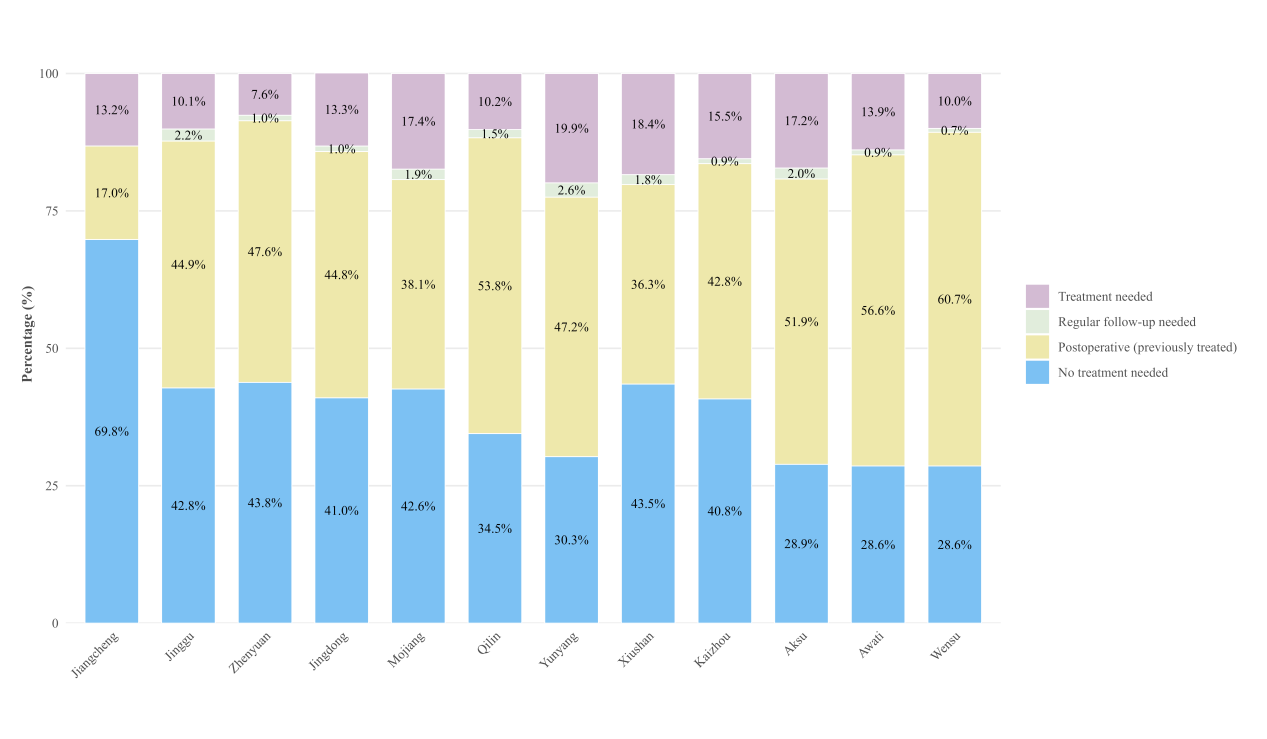


**Figure S4** Ultrasound examination recommendations for CHD childrens in different counties and cities.


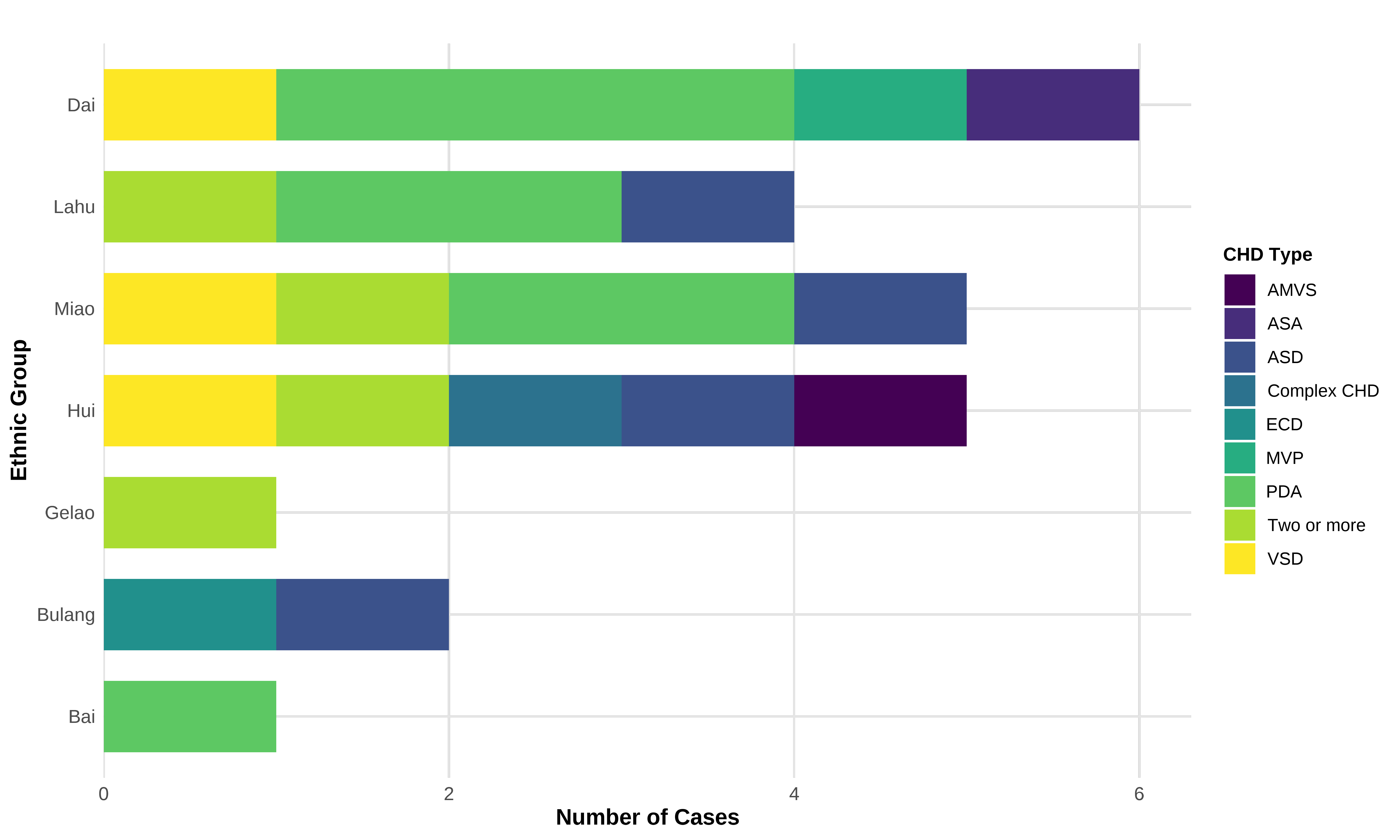


**Figure S5** Distribution of CHD types among pediatric patients requiring treatment for congenital heart disease (CHD) in different ethnic groups. This figure includes ethnic groups with fewer than ten children diagnosed with CHD.


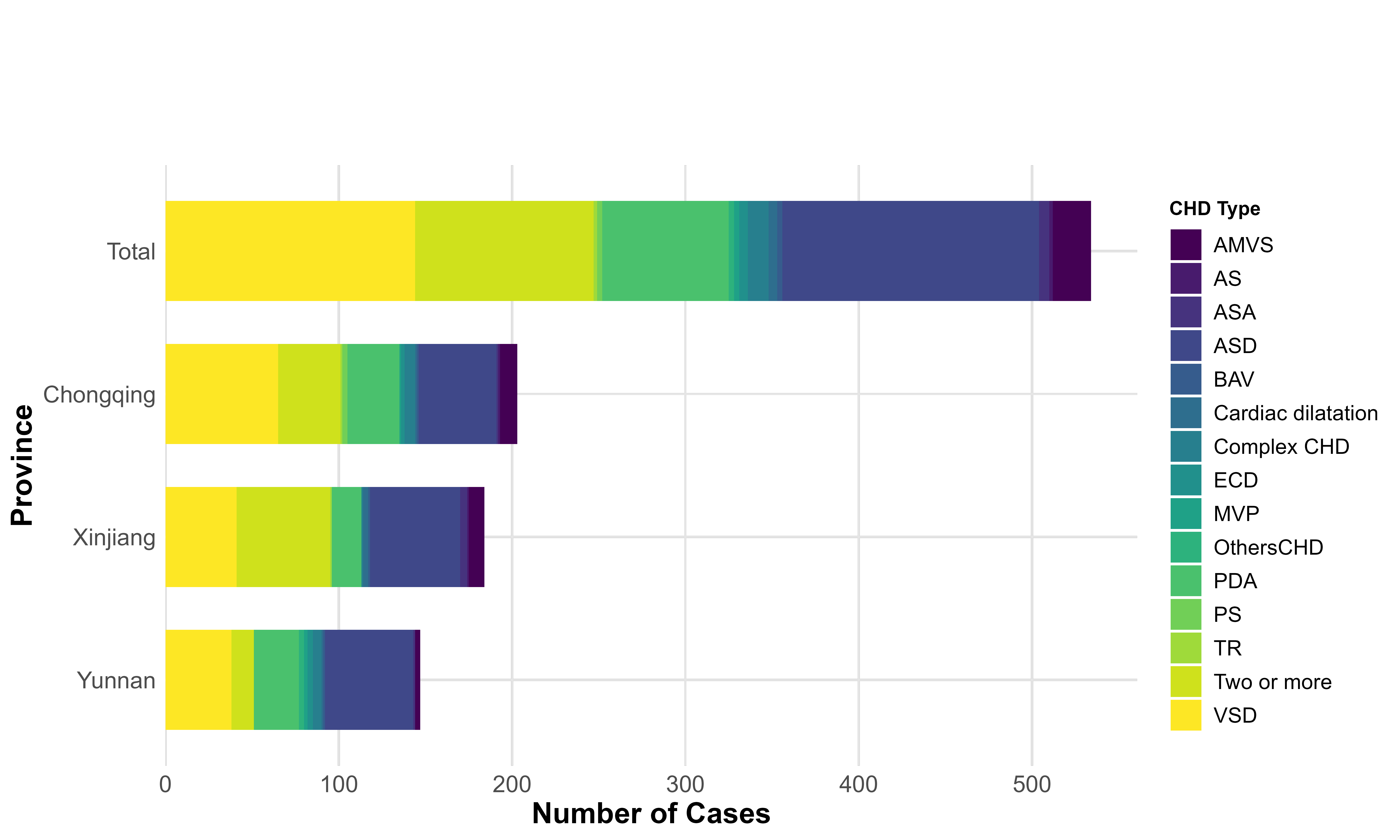


**Figure S6** Distribution of CHD types requiring treatment among pediatric patients in the three regions.


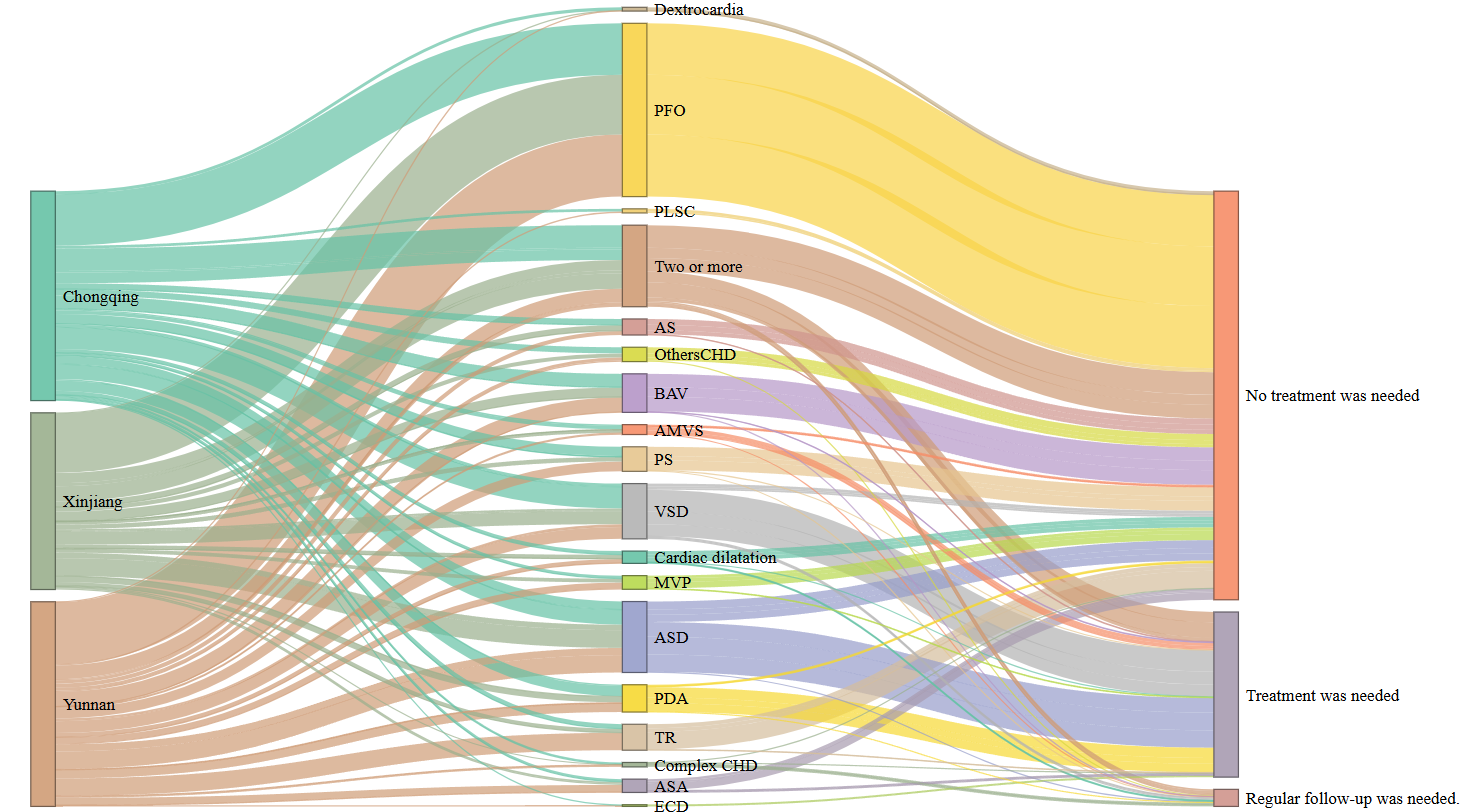


**Figure S7** CHD types and ultrasound examination recommendations flow at different regions. The Sankey diagram was applied to visualise the distribution of CHD types and ultrasound examination recommendations composition in different regions. The first column of bars indicates the region groups, the second column indicates the CHD types and the third column of bars indicates the ultrasound examination recommendation. This Sankey diagram represents the composition of the CHD types in different regions from left to right, and further flows into the ultrasound examination recommendations.


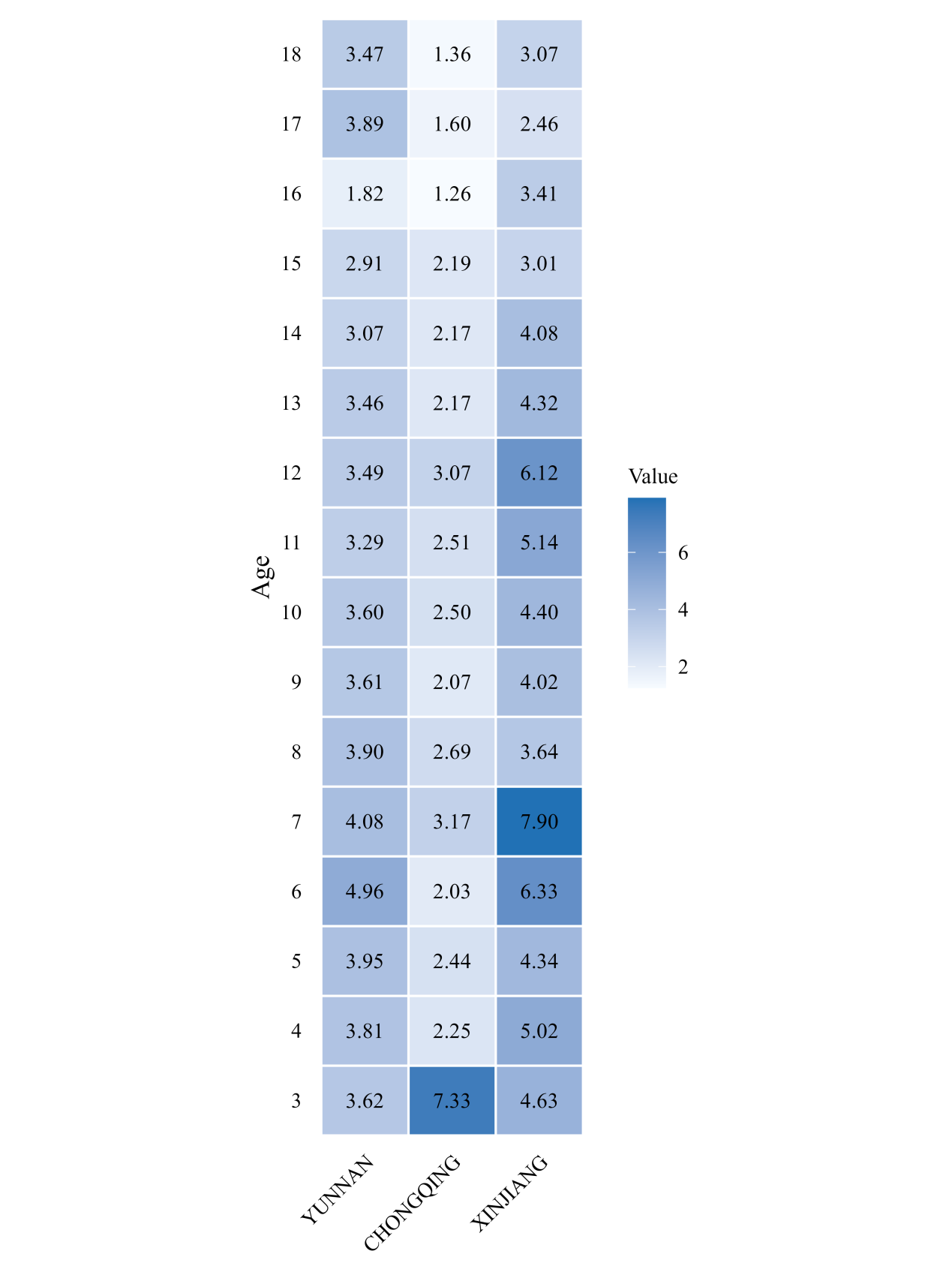


**Figure S8** Prevalence distribution of CHD by age in the three regions.


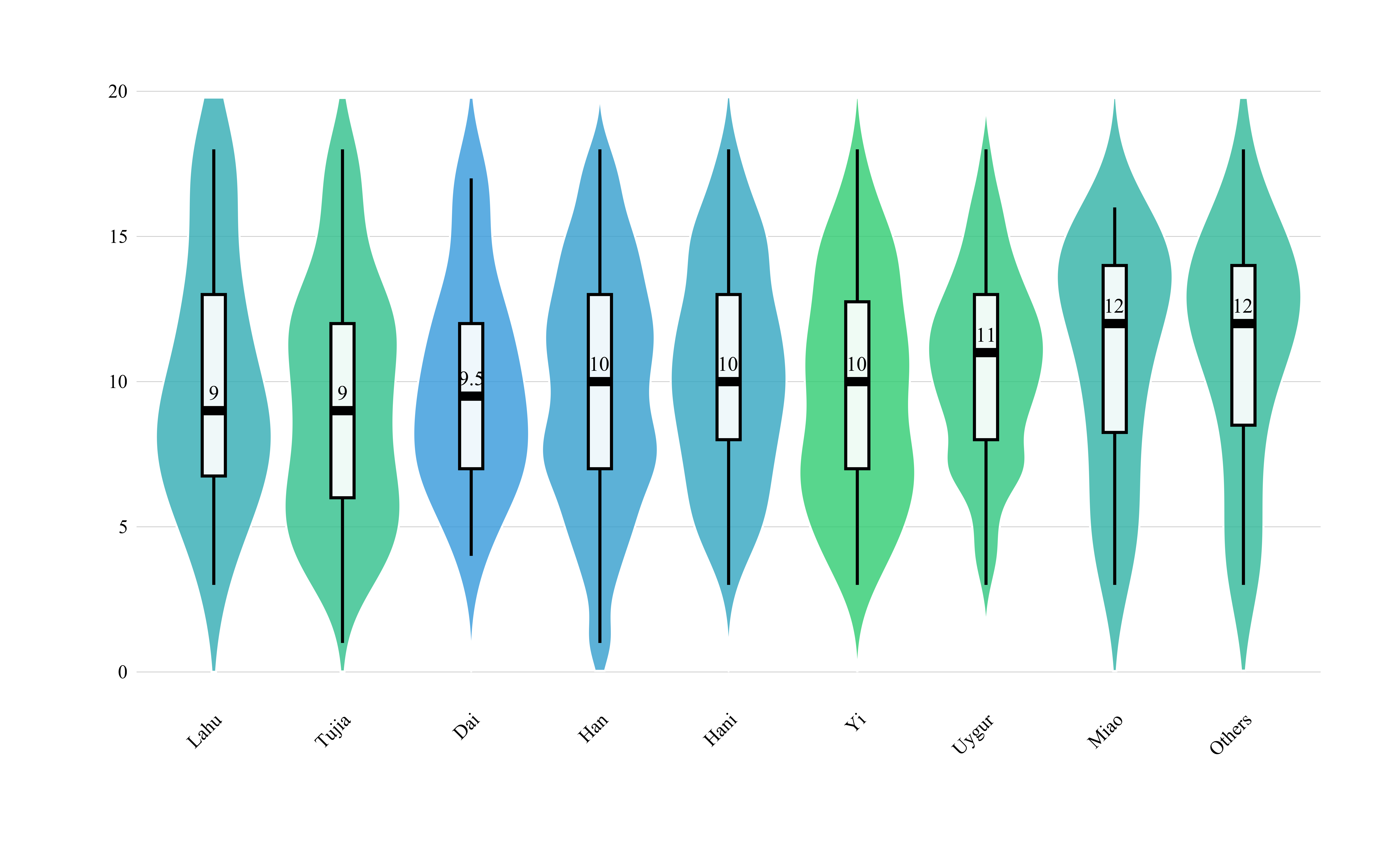


**Figure S9** Age distribution of CHD children in different ethnic groups.

**Supplementary Tables**

**Table S1** Prevalence of CHD in different screening regions.

| Province | County | Number of townships | Number of screening schools | Screening children | Confirmed case of CHD | Prevalence rate(‰) |
| --- | --- | --- | --- | --- | --- | --- |
| Yunnan | Jiangcheng | 8 | 40 | 18387 | 53 | 2.88 |
|  | Jinggu | 12 | 80 | 42281 | 122 | 2.89 |
|  | Zhenyuan | 10 | 52 | 27381 | 106 | 3.87 |
|  | Jingdong | 12 | 75 | 34442 | 120 | 3.48 |
|  | Mojiang | 16 | 77 | 40803 | 155 | 3.80 |
|  | Qilin | 14 | 318 | 155099 | 586 | 3.78 |
|  | Total | 72 | 642 | 318393 | 1142 | 3.59 |
| Chongqing | Yunyang | 15 | 216 | 141512 | 307 | 2.17 |
|  | Xiushan | 27 | 291 | 97627 | 223 | 2.28 |
|  | Kaizhou | 47 | 358 | 209317 | 542 | 2.59 |
|  | Total | 89 | 865 | 448456 | 1072 | 2.39 |
| Xinjiang | Aksu | 21 | 207 | 155152 | 540 | 3.48 |
|  | Awati | 12 | 108 | 57416 | 332 | 5.78 |
|  | Wensu | 15 | 65 | 45114 | 290 | 6.43 |
|  | Total | 48 | 380 | 257682 | 1162 | 4.51 |
| Total |  | 209 | 1887 | 1024531 | 3376 | 3.30 |

**Table S2** **Regional Heterogeneity in Socioeconomic Status, Healthcare Resources, and the Prevalence of CHD.**

|  | Prevalence  (per 1,000 people) | GDP per capita  (CNY) | Per Capita Disposable Income of Households(CNY) | Hospital beds  (per 1,000 people) | Physicians (per 1,000 people) | Percentage of Ethnic Minorities  (% of total population) |
| --- | --- | --- | --- | --- | --- | --- |
| Yunnan | 3.59 | 67612 | 29932 | 8.03 | 9.7 | 33.1 |
| Chongqing | 2.39 | 94135 | 39713 | 7.8 | 8.9 | 6.8 |
| Xinjiang | 4.51 | 78290 | 30899 | 6.5 | 8.2 | 57.8 |

|  | Total | Han | Uygur | Tujia | Hani | Yi | Dai | Lahu | Miao | Others | P value |
| --- | --- | --- | --- | --- | --- | --- | --- | --- | --- | --- | --- |
| Ultrasound findings |  |  |  |  |  |  |  |  |  |  | <0.01 |
| No treatment was needed | 1198(35.5) | 662(36.1) | 284(29.5) | 71(45.2) | 65(43.0) | 60(42.3) | 23(46.0) | 18(64.3) | 10(38.5) | 5(19.2) |  |
| regular follow-up was needed. | 50(1.5) | 27(1.5) | 15(1.6) | 2(1.3) | 1(0.7) | 2(1.4) | 1(2.0) | 0(0.0) | 1(3.8) | 1(3.8) |  |
| Treatment was needed | 484(14.3) | 246(13.4) | 139(14.4) | 34(21.7) | 26(17.2) | 18(12.7) | 5(10.0) | 4(14.3) | 4(15.4) | 8(30.8) |  |
| Postoperative (previously treated) | 1644(48.7) | 897(49.0) | 526(54.6) | 50(31.8) | 59(39.1) | 62(43.7) | 21(42.0) | 6(21.4) | 11(42.3) | 12(46.2) |  |

**Table S3** Ultrasound recommendations for children with CHD from different ethnic

Groups.

**Table S4** This study screened for the types of children with congenital heart disease and ICD-10-CM Code

| Diagnosis | ICD-10-CM Code |
| --- | --- |
| Patent foramen ovale(PFO) | Q21.101 |
| Atrial septal defect(ASD) | Q21.100 |
| Ventricular septal defect(VSD) | Q21.000 |
| Bicuspid aortic valve(BAV) | Q23.101 |
| Patent ductus arteriosus(PDA) | Q25.000 |
| Tricuspid regurgitation(TR) | I07.100 |
| Pulmonary stenosis(PS) | Q22.100 |
| Aortic stenosis(AS) | Q25.300 |
| Mitral valve prolapse(MVP) | I34.100 |
| Atrial septal aneurysm(ASA) | I25.304 |
| Cardiac dilatation | Q24.000 |
| Aneurysm of membranous ventricular septum(AMVS) | I25.300×005 |
| Persistent left superior cava(PLSC) | Q26.100 |
| Dextrocardia | Q24.000 |
| Endocardial cushion defect(ECD) | Q21.207 |
